# Is oesophageal manometry a must before laparoscopic fundoplication?

**DOI:** 10.4103/0972-9941.78357

**Published:** 2011

**Authors:** Vipul D Yagnik

**Affiliations:** Ronak Endolaparoscopy and General Surgical Hospital, Patan, Gujarat, India

Dear Sir,

I read the article entitled, “Is esophageal manometry a must before laparoscopic fundoplication? Analysis of 46 consecutive patients treated without preoperative manometry,”[1] with great interest. I would like to congratulate all authors for performing such a study on patients from developing countries, where all the facilities are not available. However, certain aspects need clarificaration. Endoscopy is typically the first test to diagnose gastro-oesophageal reflux disease (GERD). However, the two major pitfalls of endoscopy are: (1) Mucosal changes are absent in 50% of the cases[2] and (2) Interobserver variation is present, particularly for low-grade oesophagitis.[3] Looking into these pitfalls, is it justified to go for surgery only on the basis of endoscopic diagnosis? How do you decide which type of operation is indicated in particular patients? It is well known that manometry gives an idea about the propulsive force of the body of the oesophagus. Oesophageal manometry assessed the, length, location, and pressure of the LES(Lower Esophageal Sphincter), along with its ability to relax during swallowing. In addition, it also allows proper placement of the probe for ambulatory pH monitoring. Tailoring of the antireflux procedure is based on peristaltic contraction. Patients with normal peristalsis do better with a full 360 fundoplication, while patients with peristaltic failure do well with partial fundoplication. I would like to know from the authors, whether they recommend a full 360 fundoplication for all patients. Do they not recommend 24-hour pH monitoring as a pre-operative investigation? Ambulatory pH monitoring is the gold standard test for the diagnosis of GERD, with a sensitivity and specificity of about 92%.[4] It is of key importance in the workup for the following reasons. Ambulatory pH monitoring is the only way to quantitatively express the overall degree and pattern of oesophageal acid exposure, both of which may impact the decision towards surgery. It gives an idea about abnormal reflux. In the UCSF(University of California, San Francisco) study,[3] pH monitoring yielded normal results in 30% of the patients, with a clinical proven diagnosis of GERD, thereby obviating the need for the continuation of proton pump inhibitors (PPIs) or the performance of an antireflux surgery. Not only is this study is retrospective, but it is non-comparative, so we have no way to evaluate whether the suggested approach to go for surgery without manometry and pH study has any advantage over the established gold standard. I think a well-designed, randomised, controlled trial is required to prove whether manometry should be performed or not.
